# Mental health first aid in the workplace: a reflexive thematic analysis of UK workers’ experiences

**DOI:** 10.1080/17482631.2026.2706909

**Published:** 2026-07-21

**Authors:** Opeyemi Atanda, Eleni Vangeli, Steven D. Brown, Patrick Callaghan, Graham Durcan, Kerry Wood, Tim Carter, Nick O’Shea, Sarah White, Paula Reavey

**Affiliations:** a School of Allied Health and Life Sciences, London South Bank University, London, UK; b Nottingham Trent University, Nottingham, UK; c Centre for Mental Health, London, UK; d University of Nottingham, Nottingham, UK; e St George’s University of London, London, UK

**Keywords:** Reflexive thematic analysis, mental health first aid, workplace mental health, help-seeking, organisational culture

## Abstract

**Background:**

Mental Health First Aid (MHFA) is used in workplaces to improve mental health literacy, reduce stigma, and promote help-seeking. This paper explores how employees perceive and engage with MHFA in work settings.

**Methods:**

This qualitative study was embedded within the EMPOWER cluster randomised controlled trial (Atanda et al., 2020). Twenty-four participants, including Mental Health First Aiders, recipients of MHFA support, employees, and senior managers, took part in semi-structured interviews. Guided by a relativist epistemological position, data were analysed using reflexive thematic analysis.(Atanda et al., 2020).

**Findings:**

Three themes were identified: (1) ambiguity surrounding the purpose of MHFA; (2) uncertainty and scepticism regarding the role and effectiveness of MHFA (subthemes: Anticipated Stigma, MHFA not addressing core concerns affecting staff well-being); and (3) risks to personal relationships following disclosure. Participants questioned the purpose, boundaries, and organisational value of MHFA, contributing to reluctance to engage. Disclosure decisions were shaped by concerns about stigma, confidentiality, and potential impacts on workplace relationships and career progression.

**Discussion:**

The findings suggest MHFA is most effective when part of a broader organisational commitment to employee wellbeing. Clarifying MHFA's purpose, addressing workplace stigma, and tackling organisational sources of distress may improve engagement and strengthen mental health initiatives.

**Clinical trial registration:**

Clinicaltrials.gov NCT04311203. Registered on 17 March 2020, protocol published in 2020.

## Background

The aim of Mental Health First Aid (MHFA) is to improve people’s mental health literacy and encourage people experiencing mental distress to access further support and treatment services (Atanda et al., 2020). MHFA training improves Mental Health First-Aiders’ (MHFAiders) mental health literacy, increases their supportive behaviours, reduces negative attitudes and stigma, and increases MHFAiders confidence in helping a person experience mental distress to seek professional support (Hadlaczky et al., 2014; Kitchener & Jorm, 2002; Maslowski et al., 2019; Morgan et al., 2018). MHFA also appears to leave employees with a favourable view of their employers, such that this alone may improve their mental health and well-being (Kitchener & Jorm, 2002; Morgan et al., 2018; Narayanasamy et al., 2021).

The potential of MHFA to reduce stigma and lower sickness-related absence and presenteeism in the workplace speaks to wider social benefits, such as keeping people who face mental health challenges at work and reducing workplace discrimination. However, the lack of a social impact assessment of MHFA remains a significant gap in the evidence base. An increasing number of UK and overseas employers have adopted MHFA, but there has been no systematic investigation of its effectiveness on employees’ help-seeking behaviour compared with an active control. There are currently over 660,000 trained Mental Health First Aiders supporting businesses across England (Brown, 2023). A recent Cochrane Systematic Review of MHFA training trials (Richardson et al., 2023) reported that MHFA training may improve mental health knowledge and recognition of mental health problems among trainees; however, evidence regarding its impact on broader mental health outcomes of recipients (i.e. people who engaged with trainees) remains limited. This distinction is important because much of the existing evidence base focuses on outcomes among individuals who receive MHFA training, such as increased mental health literacy, confidence, and helping behaviours.

Following a review of the literature on MHFA in workplace settings, less attention has been paid to employees who might seek support from a Mental Health First Aider (referred to as recipients) and to the extent to which MHFA availability affects help-seeking behaviour in workplaces. In the EMPOWER trial, help-seeking among recipients was identified as the main outcome after consultation with funders, MHFA England. Understanding these experiences is particularly important given the increasing implementation of MHFA within organisations and the assumption that improvements in MHFAiders' knowledge and confidence will translate into improved support and help-seeking among employees.

The current study was part of the EMPOWER clustered randomised controlled trial (https://www.lsbu.ac.uk/research/centres-groups/addictive-behaviours/lived-experiences-of-distress-research-group/free-mental-health-first-aid-training) that aimed to evaluate the effectiveness of MHFA from the perspective of workplace end-users (i.e., MHFA recipients). The qualitative component (Atanda et al., 2026) was part of a process evaluation embedded within the EMPOWER cluster-randomised controlled trial. Its purpose was to explore participants’ experiences of MHFA implementation and delivery, understand how the intervention was perceived within workplace settings, and identify contextual factors that may have influenced engagement and help-seeking behaviours. Consistent with guidance on process evaluations of complex interventions (Moore et al., 2015), the qualitative study aimed to provide explanatory insight into the trial findings by examining participants’ experiences of implementation, mechanisms of impact, and organisational context. The results of the primary trial outcomes have been reported in another publication currently under review. The findings of the trial indicate that employees of organisations receiving MHFA are significantly less likely to seek formal help for their mental health difficulties. The uncertainty about the purpose of MHFA might explain the preference of employees to seek help from alternative means instead of seeking support from a mental health first aider trained within their organisation. However, MHFA was reported to improve the self-efficacy knowledge of employees in organisations where MHFA was introduced. Additionally, MHFA is reported to reduce sickness absences, yet few employees seek help from mental health first aiders despite reporting high levels of work-related mental distress and significant levels of diagnosed mental illness.

The present study was not designed to test any specific organisational theory. However, theories that distinguish between individual and organisational determinants of employee wellbeing may offer useful perspectives for interpreting workplace mental health initiatives. One such framework is Herzberg's Two-Factor Theory, which differentiates between workplace conditions that contribute to dissatisfaction and factors that promote positive experiences at work. This framework is briefly introduced here as a potentially useful interpretive lens for understanding the relationship between MHFA and the wider organisational context in which it is implemented.

## Methods

The qualitative study formed an embedded explanatory component of the EMPOWER cluster randomised controlled trial. Consistent with a mixed-methods explanatory approach, qualitative interviews were conducted to explore participants’ experiences of MHFA and to provide contextual insight into the trial findings. Particular attention was given to understanding how employees interpreted the purpose of MHFA, factors influencing engagement with MHFA support, and workplace conditions that facilitated or constrained help-seeking behaviour.

### Participants

Twenty-four participants took part in semi-structured interviews. They were purposely selected from a list of volunteers who responded to the online survey distributed during the larger trial. Participants were recruited from organisations involved in the EMPOWER trial, representing various employment sectors across England. These included charities supporting vulnerable groups, health and social care providers, independent care services, educational and community organisations, consultancy firms, publishing companies, forensic and assessment services, and public relations agencies. The organisations ranged from small (*n* = 4) and medium (*n* = 2) enterprises to larger national entities (*n* = 3). To ensure diverse insights, researchers included staff from all organisational levels. Participants were those who responded to the survey and expressed willingness to discuss their experiences of mental health and well-being. This included individuals with direct MHFA involvement (recipients, MHFAiders, or senior managers implementing MHFA) and those with indirect exposure, such as employees in offices where MHFAiders are based. Purposive sampling targeted stakeholder groups likely to provide different perspectives on MHFA's implementation, including MHFAiders, recipients, employees, and senior managers. Recruitment took place within EMPOWER trial organisations and continued throughout the data collection period. The final sample depended on participants’ availability and willingness. Notably, few employees received MHFA support, and only one senior manager agreed to participate, resulting in some groups being underrepresented compared with the plan. Aligned with a reflexive thematic analysis (Braun & Clarke (2021), recruitment was not driven by a set notion of data saturation but aimed to gather diverse experiences across stakeholder groups, considering the practical recruitment challenges within the trial.

The breakdown of participants interviewed was a) MHFA recipients (*n* = 5), b) mental health first aiders (*n* = 10), c) employees of the organisation where MHFA was introduced and who did not engage with MHFA (*n* = 8), and d) a senior manager (*n* = 1) involved in the implementation of the intervention. Four of the five intervention recipients interviewed were also trained mental health first aiders but were not part of the 10 mental health first aiders interviewed. The overlap between participant roles was explicitly considered during analysis. Rather than assigning participants to a single stakeholder category, we attended to the experiential context of each account. Where participants discussed their experiences of receiving support, these narratives informed the recipient analysis; where they reflected on their experiences as trained MHFAiders, these accounts informed our interpretation of implementation and delivery. Throughout theme development, the research team reflected on how participants' dual roles might shape their interpretations of MHFA and considered this in the development of themes.

### Data collection

Interviews were conducted between May and November 2021, a period during which UK organisations were transitioning from COVID-19 restrictions towards more flexible working practices. Participants represented organisations operating across office-based, remote, and hybrid working arrangements. These differing working environments formed an important context for participants' experiences of MHFA, as opportunities for informal contact with colleagues, visibility of Mental Health First Aiders, and access to workplace support were likely to differ depending on working arrangements. As such, participants' accounts should be interpreted within the context of evolving post-pandemic workplace practices. All Interviews were conducted online using video conferencing software, with interviews lasting an average of 60 minutes. The interviews were audio-recorded and transcribed verbatim. Interviews were semi-structured and constructed with a specific set of themes derived from gaps in the literature and theoretical directives. However, sufficient flexibility in the interview structure was ensured to prompt participants to elaborate further on their experiences, representing a greater depth of response. In a systematic methodological review of qualitative studies (Kallio et al., 2016), five phases were outlined to develop an interview guide. The phases included: 1) identifying the prerequisites for using semi-structured interviews, such as thinking about the purpose of the research; 2) retrieving and using previous knowledge and experience about the subject; 3) formulating a preliminary semi-structured interview guide; 4) pilot testing; and 5) presenting the complete semi-structured interview guide. The lead author (OA) developed the guide with several iterations following regular review with the co-authors of this paper (PR, KVW, EV, SDB, and PC). The interview guide was pilot tested with four individuals, including a trained MHFAider, a recipient of MHFA support, and an employee working within an organisation where MHFA had been implemented. The pilot confirmed that the questions were clear and appropriate, and therefore no revisions were required. A copy of the semi-structured interview guide is provided in “Supplementary Material”.

### Ethical considerations

The lead author’s University Ethics Committee approved the study. Participants were debriefed upon completion of the interviews, and any identifiable information was removed from the transcripts. Pseudonyms were assigned to the participants, and the original voice recordings were deleted. Finally, participants were emailed with information regarding mental health and wellbeing support services should their involvement in the study cause them distress.

### Procedure

Participants were purposively sourced from a pool of individuals who volunteered to be interviewed after completing the online survey at baseline and post-intervention. An information sheet regarding the aims of the study was sent to participants and written and verbal consent was obtained. All interviews were conducted online via Microsoft Teams and audio recorded using an external digital recorder. All participants were debriefed at the end of the interview, both in written form and verbally.

### Data analysis

The researchers used reflexive thematic analysis (RTA) to analyse the collected data. RTA involves interpreting the data while acknowledging the researcher's subjective perspective (Braun & Clarke, 2021). RTA allows the researcher to choose a theoretical framework that fits their approach to data analysis, which in the present study was a relativist perspective. The relativist perspective, which holds that realities are multiple, socially and experientially based constructions that people develop and revise in relation to context and interaction (Braun & Clarke, 2021). Within this framework, we treated coding as an interpretive practice and developed themes as meaning-based stories that were generated through iterative engagement with the dataset. Themes and subthemes presented are analytic propositions about patterned meaning in workplace MHFA experiences, rather than as definitive representations of a singular underlying reality.

The RTA involved transcribing all 24 interviews, reading them through several times, and noting initial observations or reflections. The interviews were analysed by identifying and labelling specific features relevant to the research question. The authors acknowledged that the creation of meaning was influenced by the context in which it occurred, and the researcher's subjectivity in the shaping of the analytical process. Hence, the researcher is considered to have an active role in knowledge production (Braun & Clarke, 2021). Codes were generated through the systematic labelling of segments of text that captured meaningful features of participants’ accounts. The first author (OA) conducted the primary coding of all transcripts using NVivo as part of his PhD. Coding was an iterative process involving repeated engagement with the data and the development of both descriptive and interpretive codes. The coding was primarily inductive, which allowed themes to be observed from the data itself, ensuring that participants’ experiences shaped the analytic process rather than being constrained by predefined categories. To enhance reflexivity and analytical depth, regular discussions were held with four members of the research team throughout the analytic process (EV, PC, PR, and SDB). These discussions focused on exploring alternative interpretations of the data, challenging assumptions, examining relationships between codes, and refining the developing thematic structure. Rather than seeking coder agreement, these conversations were used to deepen engagement with the data and support the development of themes that captured patterned meanings across participants' accounts. Through this iterative process, initial codes were progressively organised into candidate themes and subsequently refined into the final thematic framework. Candidate themes were subsequently identified and iteratively refined by mapping them back to the research aims. For example, codes related to uncertainty about the purpose of MHFA, confusion about the role of Mental Health First Aiders, and ambiguity about available support pathways were initially treated as distinct categories. Through discussions within the research team, these codes were interpreted as reflecting a broader pattern of ambiguity regarding the purpose of MHFA. Similarly, codes relating to anticipated stigma, concerns about disclosure, personal reservations about sharing difficulties, and perceptions that MHFA did not address underlying workplace stressors were subsequently integrated into a higher-order theme reflecting participants' ambivalent engagement with workplace mental health support. This process enabled the analysis to move beyond descriptive categorisation towards the identification of broader interpretive themes. We have included the concepts and project maps developed on NVivo, showing the development of the themes in the supplementary material. In the final stage, themes were reviewed and clearly defined, merging overlapping candidate themes and ensuring conceptual coherence.

### Reflexivity statement

The research team recognised that their training and professional identities provide lenses through which data are interpreted rather than neutral filters. It is important to recognise the influence of the researchers' backgrounds in shaping the study’s approach and interpretation (Braun & Clarke, 2021). The lead authors OA, PC, PR, EV, KVW, and GD were all trained as MHFAiders as part of the EMPOWER project, which provided them with first-hand experience and a deeper understanding of the principles, processes, and practical applications of MHFA. This prior training encouraged a more nuanced engagement with the data, allowing for insights that may not have been as easily accessible to researchers without similar experience. While this familiarity provided insight into the intended aims and the operational definition of MHFA, it also created the potential for confirmatory bias, particularly in interpreting participant accounts as evidence of the intervention's effectiveness. We engaged in critical reflection was embedded throughout the analytic process rather than treated as a discrete stage of analysis. The first author undertook the primary coding of all transcripts, while four additional authors participated in regular analytic discussions in which emerging codes, themes and interpretations were critically examined.

These discussions involved us actively questioning how our prior knowledge of, and familiarity with, MHFA may have shaped our interpretations of the data. For example, beyond being drawn to participants' descriptions of MHFA as increasing awareness, providing emotional support and facilitating conversations. Through team discussions, we became aware that this emphasis may have reflected our own expectations that MHFA would be experienced positively. However, subsequent discussions prompted greater attention to participants' expressions of scepticism, reluctance to engage, concerns about confidentiality and disclosure, and perceptions that MHFA addressed symptoms rather than the organisational causes of workplace distress. Engaging with these accounts prompted us to reconsider our initial analytic direction, and rather than positioning them as peripheral or exceptional, we incorporated them as central to the development of our final themes. This process enabled us to produce a more nuanced interpretation that reflected both the perceived benefits and limitations of MHFA.

A relativist ontology asserts that there are multiple realities and that individual experiences are socially constructed and situated in time, context and social interaction (Braun & Clarke, 2021). Rather than treating interview transcripts as fixed facts, we approached them as co-constructed narratives shaped by both participants and researchers. Our training and prior understanding of the literature around workplace mental health culture enriched our reading of these narratives. We had regular analysis meetings where we challenged our beliefs and interpretations whilst ensuring that interpretations were context-bound.

### Findings

**Figure 1.**
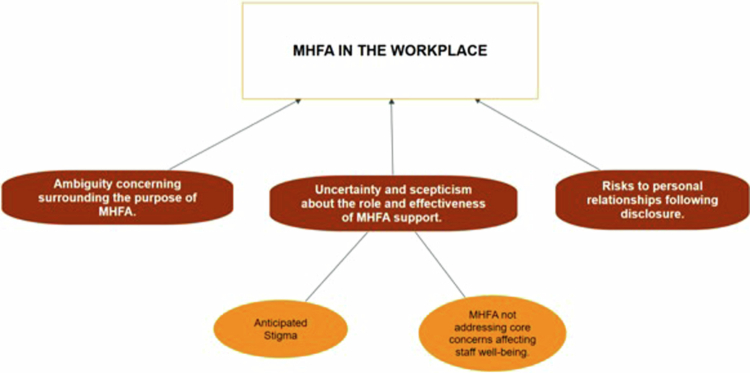
Thematic map illustrating employees' experiences and perceptions of Mental Health First Aid (MHFA) in the workplace.

Following the analysis, three main themes and two subthemes related to the experiences of both direct and indirect recipients of MHFA in the workplace were apparent. Participants were asked questions about their understanding of their workplace engagement experience with MHFA. Below is a thematic map in Figure 1 that shows the relationship between the themes and subthemes

***Theme 1: Ambiguity surrounding the purpose of MHFA***

This theme captures participants' uncertainty regarding the intended purpose and scope of MHFA within workplace settings. Although many participants viewed MHFA positively, their accounts suggested that uncertainty about whether MHFA functioned as a listening service, a signposting mechanism, a wellbeing initiative, or a route to organisational support shaped how they engaged with the intervention. This ambiguity appeared to limit confidence in the intervention and contributed to inconsistent expectations regarding the support MHFA could realistically provide.

Previous research on workplace mental health and wellbeing interventions consistently identifies staff engagement as a key determinant of implementation success. Studies indicate that low levels of engagement, such as limited awareness of available resources, reluctance to participate, or concerns about stigma, can reduce the uptake and effectiveness of interventions (Joanna et al., 2022; Mellor & Webster, 2013; Quirk et al., 2018). The lack of understanding about the purpose of MHFA contributes to a lack of motivation or personal responsibility, which has been reported as a potential barrier to staff engagement in workplace well-being initiatives (Quirk et al., 2018).

As “Recipient 2” highlighted, there appears to be a shared view that MHFA could serve as an improvement compared with previously introduced mental health initiatives. Previous initiatives were perceived to be tokenistic, tick-box exercises aimed to publicly present the organisation in a positive light.

“*And I guess for me, this sort of sits in between. Certainly has provided a better framework for the conversation rather than, I don't know... The employees, since moving offsite, sort of felt like the response prior to this was, “Oh, yeah, we have a helpline for that. Or oh, there's an app, download Headspace”, you know. I think the response from a lot of organisations is, oh, yeah, we have an app for that, as an app can fix your well-being. I mean, it is lovely to have a meditation app. It is lovely to have well-being and yoga sessions, but I am not really addressing those mental health issues that people have and how that affects them in the workplace. For me, this is, I guess, more meaningful; it feels a little bit like we are having more meaningful conversations about it.* (Recipient 2)

Recipient 2 acknowledges that existing individually focused interventions such as a meditation app or yoga sessions, are not unwelcome, but do not adequately address mental health issues. The introduction of MHFA is an improvement, which is ‘more meaningful’. However, there is still a feeling of a lack of clarity about the purpose of MHFA, the roles of the mental health first aiders, and the initiative's purpose as a support tool within the organisation. As some employees expressed, this ambiguity regarding purpose was perceived to represent a substantial barrier to engagement.

“*I do not know if it provides the level of continuity that people need to have this conversation with the same person.”* (Employee 8)

“*I guess it was maybe a lack of understanding on my part about the role of the Mental Health First Aider and what support they can give us?”* (Employee 5)

Similarly, trained mental health first aiders interviewed as part of the study discussed the perception of MHFA as a corporate initiative to make people work more efficiently rather than improve mental health per se (see extract from participant “First aider 8” below). The need for clarity of purpose for employees appears to be a key focus for reducing their ambiguity. The feeling was not that MHFA is entirely good or bad, but rather that there was suspicion regarding how it was being deployed and what the managers' aims were in doing so:

*“Many people may see this as a corporate initiative rather than a Mental Health First Aid initiative.*(First aider 8)*”*

*“There may be some suspicion about the intent of Mental Health First Aid in a corporate setting. Is it there to make people better workers? Is it there really to support people? Is the company monitoring these conversations? You know, I know it sounds… (First aider 8)”*

These accounts suggest that uncertainty was not simply a lack of awareness of MHFA. Rather, participants appeared to hold competing understandings of what MHFA was intended to achieve. As a result, some employees struggled to determine when MHFA should be used and whether it represented an appropriate avenue for support compared with existing organisational or external resources.

In addition, there was a lack of understanding of the capabilities of mental health first aiders to address employees’ workplace issues, which contributed to their mental health difficulties. During discussions about MHFA and mental health initiatives in the workplace, other factors contributing to the reluctance to engage with MHFA were also reported; these are detailed below.

***Theme 2: Uncertainty and scepticism about the role and effectiveness of MHFA support***

The uncertainty surrounding the purpose of MHFA in the workplace contributed to a hesitancy around seeking help from mental health first aiders. There is a mix of scepticism, uncertainty and concerns regarding the effectiveness and appropriateness of MHFA support in the workplace. When the first aiders were asked about colleagues seeking support from them, participants reported a lack of understanding of their roles and of the practice of MHFA as contributing to a lack of engagement.

*“But when I was talking to some colleagues in a separate team meeting, and they are asking me about what I do as part of that role, explaining it to them. Nearly all of them were saying, well, if we were having a problem, why would we? Why would we talk to somebody at work about it.”*(First aider 5)

This account suggests that uncertainty was not simply about awareness of the MHFA role but about its perceived legitimacy within the workplace. Participants questioned why a colleague, rather than an established healthcare professional or manager, would be an appropriate source of support for personal mental health difficulties. In this sense, engagement was influenced by employees' interpretations of the role and whether it was perceived as a credible and appropriate source of help.

Participants also questioned whether organisations genuinely supported openness about mental health. Although MHFA was intended to facilitate conversations about wellbeing, some participants were uncertain whether disclosure would be accepted or whether it might have unintended consequences for how they were perceived within the workplace.

As expressed by a MHFAider, it appears people's reluctance might be linked to their lack of appreciation that it is acceptable within the organisation to be open about mental health struggles:

*“I do not think it is very clear to people that you can disclose what you are suffering with a colleague and that you are going through a bit of a rough time, and it might take you a few months to get back up on your feet.”* (First aider 5)

Rather than reflecting confidentiality concerns alone, the above account suggests that participants were evaluating whether their workplace was psychologically safe enough to disclose mental health difficulties. Decisions about engaging with MHFA therefore appeared to involve assessing both the availability of support and the anticipated social consequences of disclosure. This interpretation aligns with research suggesting that mental health difficulties often represent a concealable stigma, where individuals carefully consider the interpersonal and professional consequences of disclosure before seeking support (King et al., 2021). Similarly, concerns regarding discrimination and negative reactions from colleagues have been identified as significant barriers to workplace help-seeking (Brouwers et al., 2020).

A reluctance to engage was reported to be connected to negative responses encountered by other employers and managers following direct experience of mental health disclosures. As “employee 6” highlighted in the extract below, the lack of engagement is fuelled by individuals' lack of clarity regarding the role of the mental health first aider and the support they may be able to offer. The lack of understanding regarding the “powers" that MHFAiders possess to resolve issues seems to be a key factor here. This also relates to workplace systems that act as barriers to the potential benefits of engaging with MHFA support, often evident through unclear communication and guidance on how employees can utilise the MHFA resources. Additionally, the absence of an inclusive and understanding environment may perpetuate fear or shame among those seeking help.

*“What happens next to kind of resolve this issue? And I think that is the problem. I do not feel like necessarily when people are under pressure that it is necessarily resolved.”* (Employee 6)

*“If I am going through the problem, I still do not have clarity on how that is resolved within the company, I guess.”* (Employee 6)

There appears to be a perception that disclosure to a MHFAider will not trigger further mechanisms within the organisation that might help directly resolve the problem. Participants' engagement with MHFA was shaped by a complex balance between perceived benefits and perceived risks. Although many participants valued the availability of support, concerns regarding disclosure, confidentiality, stigma, and organisational motives often moderated their willingness to seek help. Importantly, reluctance to engage was rarely indicative of opposition to MHFA itself. Rather, participants appeared to be evaluating the broader social and organisational consequences of disclosing mental health difficulties within workplace settings. Whilst the systemic issues act as an engagement barrier, anticipated stigma from perceived reactions to mental health disclosures will be discussed as a subtheme, as this further added to anxiety over using MHFA and contributed to a reluctance to engage.

**Subtheme: anticipated stigma**

Participants frequently expressed reluctance to engage with MHFA due to anticipated stigma. Anticipated stigma is an expectation of some humiliating experience related to engaging with MHFA. It is defined as the "extent to which individuals are concerned about being the target of stereotyping, prejudice and discrimination" (Fox et al., 2016p. 883). Research on people living with chronic illness has reported anticipated stigma to be a predictor of avoidance and underutilisation of needed care (Earnshaw et al., 2012). Participants discussed the contribution of anticipated stigma concerning their reluctance to seek support, which can be linked to perceived public stigma towards mental health difficulties in general. (Quinn et al., 2014).

*“I just do still wonder whether there is a bit of a stigma about asking for help internally. And I think that could be a barrier”.* (Employee 4)

*“And I would say it's not the lack of the team trying and supporting you, but it is more my mentality that I do not necessarily want people at work to know how I am necessarily feeling.”* (Employee 6)

A common concern arose whenever employees felt the need to share their mental health difficulty; reactions and anticipated steps taken by a mental health first aider after seeking support appeared to augment this fear. As reported by one participant (First Aider 5):

*“I think people worry about, well, if I say I've got this, or if work finds out that I'm dealing with this right now. Or if work finds out that I'm really stressed with a workload and I'm going for this promotion, I'm not going to get that promotion, or they're going to think I'm incapable of doing my job.”* (First Aider 5)

Participants reported holding back on sharing their difficulties if they were applying for promotion or moving jobs/roles within the organisation, as they suspected they would be regarded as ‘incapable of doing the job’. This phenomenon is not far from the already known public stigma surrounding mental health difficulties. Research into public attitudes still indicates strong negative stereotypes, including perceptions that individuals with such difficulties are unpredictable, aggressive, violent, dangerous, unreasonable, less intelligent, lacking in self-control, and frightening (Hampson et al., 2020). Moreover, previous studies have reported a link between the mental health disclosure of workers and stigma-related negative responses from managers and colleagues (Brouwers et al., 2016; Corrigan & Matthews, 2003). Participants discussed instances where they had considered disclosing their mental health concerns but decided against doing so due to anticipated stigma.

Participants' accounts suggest that anticipated stigma remained an important barrier to workplace help-seeking despite increasing awareness of mental health issues. Concerns about being judged, perceived differently, or viewed as less capable contributed to cautious decision-making regarding disclosure. These findings indicate that improving access to support alone may be insufficient if employees continue to perceive mental health disclosures as carrying professional or social risks.

***Subtheme: MHFA not addressing core concerns affecting staff well-being***

Another subtheme concerned participants' perceptions that MHFA did not address the underlying organisational factors contributing to poor mental health. Rather than questioning the value of emotional support itself, participants frequently distinguished between interventions that helped employees cope with distress and organisational action capable of addressing the conditions giving rise to that distress. Workload, staffing pressures, competing demands and organisational expectations were commonly described as persistent stressors that remained unchanged despite access to MHFA support.

As one recipient reported:

*“This is what I meant earlier on about like his mental health first aid treating stuff symptomatically; the cause has not gone away; the mountain of work is still going to be there when you get back. And if that is one of the things that is stressing you out, I have turned up, and I have got 101 different things to do. The stress is getting to me, and it makes me feel anxious and terrible. I need to like to control my panic attacks; I need to deal with that kind of thing. I am going to take the day off. I am a line manager and mental health first aider. Cool, good, go for it, and look after yourself. You go away, and you come back the next day; it is the same; the work has not changed.”* (Recipient 1)

This account illustrates that participants viewed MHFA as operating primarily at the level of symptom management rather than organisational change. The participant acknowledged the value of receiving support to manage anxiety and panic attacks but questioned whether this support could meaningfully influence the workplace conditions responsible for generating distress. The perceived benefit of MHFA was therefore viewed as temporary, enabling employees to cope with immediate emotional difficulties without altering the organisational demands that continued to undermine their wellbeing.

There seems to be a disconnect between the support that mental health initiatives like MHFA provide and any organisational level management of the core concerns perceived to detrimentally affect employees' mental health. Although there was recognition that employers issued regular surveys to flag issues affecting staff member's mental health, they were not perceived to have any positive effect on working conditions and related mental health issues. As a result, employees reported having lost confidence in raising concerns that would not be taken seriously, addressed or resolved;

*“Well, like these pulse surveys, they are anonymous employee surveys, and we have given feedback through them regularly saying there are problems. And the results will come out, and they will go, oh, look, there is a problem. And then there is no follow-up, there is nothing done on it until the next survey, and then they are surprised that the problem has not gone away.”* (Employee 4)

*“If they are really serious about somebody's mental health, it is about making sure that we have that support mechanism of resources around individuals.”* (Employee 8)

These accounts suggest that participants distinguished between organisations acknowledging employee concerns and organisations acting upon them. Rather than criticising the existence of wellbeing initiatives, participants questioned whether they resulted in meaningful organisational change. Repeated experiences of providing feedback without observing tangible improvements appeared to diminish confidence that raising concerns would lead to action. Consequently, participants evaluated MHFA within this broader organisational context; where existing wellbeing initiatives were perceived as symbolic rather than responsive, MHFA was similarly interpreted as another initiative with limited capacity to influence employees' working conditions. This interpretation aligns with evidence that organisational resources and leadership commitment play a central role in shaping employee wellbeing and workplace performance (Nielsen et al., 2017), and that employee surveys alone are unlikely to improve wellbeing without visible organisational action (Björklund et al., 2007).

An employee who works in HR reported the insignificance of the efforts to improve well-being given the leadership of the organisation do not address the root cause of the problem.

*“We are talking about well-being being very important; my team are helping bring in benefits to help support people, like access to Headspace meditation apps and all the rest of it. But we are not getting down to the root of the problem, which is just the way our work is organised at the moment and the expectations of everyone that's been placed on teams by the senior management, so there's not a recognition that there is a problem or that it is their responsibility to address the problem.”* (Employee 4)

Importantly, participants did not describe a rejection of wellbeing initiatives themselves but questioned whether responsibility for mental health had shifted disproportionately towards individual employees. While organisations were perceived to invest in wellbeing resources, participants frequently contrasted these initiatives with the continued presence of organisational practices that contributed to stress. This distinction suggests that participants evaluated organisational commitment not by the availability of wellbeing programmes but by whether leadership was willing to address the structural conditions generating distress. In this sense, MHFA was viewed as valuable but insufficient when implemented in the absence of broader organisational change.

In this study, when MHFAiders were asked about the common issues intervention recipients approached them for, they reported that:

*“Some of the pressures and issues that we have is that we cannot deal with face-to-face clients. And sometimes, if you have got a lot going on with COVID feeling isolated and feeling under the pressure, it has had an impact on our staff with, sometimes it taken us a lot longer to get work completed for the client, because people are working from home, it has taken a lot more time. The clients are frustrated, because they can't just walk in somewhere, they can't get the money, which puts a lot of pressure on their colleagues, due to the fact it's bombarded with telephone calls this needs doing, I can't get hold of this person, it's taking a lot more time.”* (First aider 1)

*“So, some of those phone calls with my manager would be offloading about my children or arguing about personal life. And she has said to me, you need to take a weekend away for yourself. And I would never have done that before.”* (First aider 1)

*“It's all very personal stuff that she's got going on around IVF, and her relatives have been very ill.* *”*(First Aider 3)

*“I would say most of the traffic that comes my way is to do with burnout and work stress (T).”*

*“Relationship issues.”* (First aider 8)

The range of issues described by MHFAiders highlights the breadth of concerns for which colleagues sought support. Participants described conversations encompassing both personal difficulties, such as relationship breakdown and fertility treatment, and work-related challenges including burnout, workload and isolation associated with remote working. This suggests that employees did not necessarily distinguish between personal and occupational sources of distress. Instead, wellbeing was experienced as the product of interacting personal and workplace circumstances. Consequently, MHFAiders were frequently required to respond to concerns that extended beyond the formal boundaries of the workplace and, in some cases, beyond the scope of the training it.

Rather than seeing MHFA as ineffective, participants seemed to question whether it was being asked to solve issues that stemmed elsewhere in the organisation. Their feedback reflected not only their views on the intervention but also their expectations of organisational responsibility. Many participants differentiated between support aimed at helping employees manage distress and actions targeting the root causes of that distress. Commonly cited sources of stress included workload pressures, staffing issues, organisational expectations, and workplace culture. As a result, some saw MHFA as addressing the symptoms of workplace stress rather than its underlying causes. This distinction explains why participants could value MHFA as a helpful resource while remaining sceptical about its ability to enhance overall organisational well-being.

Reflecting on the MHFA training, first aiders discussed the need for the training to address more of the day-to-day mental distress people encounter rather than MHFA’s current focus on mental health crises and official diagnoses.

*“There's one thing about the course that I thought wasn't, could have been a little bit better. And that was when it was talking about people with severe mental health problems, particularly people in psychotic crises. And I think when it gets to the talking about people who were acutely mentally unwell, that was perhaps not quite enough emphasis on the need to get professional help, in same way, that if you did, in the same way that on a physical health first aid class, there will be a very clear message about what point you need to get an ambulance. I think the Mental Health First Aid course was blurred a little bit because certain presentations are emergencies and urgent, and you must get professional help. And I think that was my only thing that I felt the course didn't say, at this point, you know, you can, it's about managing the emergency and getting help. It's not. It's beyond the point of view of supporting this person, you can keep them calm before they get help. So, I feel that in line with physical health first aid, it should be clear about what an emergency is, and getting help”* (First aider 6)

Importantly, scepticism held was not always directed towards MHFA itself. In several accounts, scepticism appeared to stem from broader experiences with workplace wellbeing initiatives perceived as symbolic rather than transformative. Participants therefore evaluated MHFA not only on the basis of individual interactions with first aiders but also in relation to organisational willingness to address the underlying sources of workplace stress. In this sense, engagement with MHFA was shaped by wider perceptions of organisational commitment to employee wellbeing.

Participants' reflections also highlighted uncertainty regarding the boundaries of the MHFA role during mental health crises. Rather than expressing a lack of confidence in the training overall, participants questioned where the responsibilities of a MHFAider ended and those of clinical services began. This uncertainty reflected broader concerns about role legitimacy and accountability, particularly when individuals presented with acute distress. Participants therefore viewed clearer guidance regarding escalation and referral as essential to ensuring that MHFAiders did not assume responsibilities beyond their competence.

These concerns were further compounded by the nature of mental health difficulties themselves. Unlike many physical health emergencies, signs of psychological distress are often not immediately observable, and individuals may actively conceal their experiences because mental illness remains a concealable stigmatised identity Quinn & Chaudoir, 2009).

A trained first aider reported this as an unsatisfactory and unrealistic aspect of the training.

*“And I felt like the training focused a lot on, you know, people's actions, and, you know, things that they might say, but also their behaviour. And obviously, you can't observe any of that behaviour or, you know, to a very limited extent.”* (First aider 2)

Rather than questioning the value of MHFA training itself, this account highlights a perceived mismatch between the assumptions underpinning the training and the realities of workplace practice. Participants recognised that MHFA training encourages first aiders to identify behavioural and verbal indicators of distress, yet they questioned the feasibility of this expectation in working environments where colleagues may have limited day-to-day contact or actively conceal their difficulties. Consequently, uncertainty centred not only on recognising signs of distress but also on knowing when observations were sufficient to justify intervention or escalation.

This perceived ambiguity extended the responsibilities of MHFAiders beyond providing support to making judgements about the presence and severity of mental health difficulties. For some participants, this created a sense of responsibility that exceeded the knowledge and confidence they felt the training had equipped them to manage, particularly where there was potential risk of serious harm or suicide. Rather than suggesting that MHFA is ineffective, these accounts indicate that participants viewed clearer guidance on recognising the limits of the role and the threshold for referral to professional services as an important component of effective implementation. More broadly, the findings suggest that concerns about role boundaries and accountability may themselves become barriers to confidence in delivering MHFA support.

Taken together, these findings suggest that barriers to engagement with MHFA extended beyond concerns about stigma or awareness alone. Participants' willingness to engage with support was influenced by their interpretation of the relationship between MHFA and the broader organisational environment. Where MHFA was viewed as part of a genuine commitment to employee wellbeing, participants described greater openness to engagement. Conversely, where organisational stressors remained unchanged, MHFA was sometimes perceived as addressing the consequences of distress rather than its underlying causes.

***Theme 3: risks to personal relationships following disclosure***

The risks of disclosure intersecting with interpersonal relationships were observed as a contributory factor to MHFA engagement. Some employees reported that their hesitation to engage with Mental Health First Aid was due to the potential impact on interpersonal relationships at work. Coping with the aftermath of the disclosure was quite prominent, as well as not feeling ready to bear the consequences of people knowing about their mental health issues.

*“I think I could see where there would have been opportunities that I could have shared this, but I think I chose not to, I think, largely because I think with all these sorts of mental health things, you have to feel ready to cope with what comes back, if that might make sense, which would all be very positive I'm sure and helpful, but I kind of thought about it a number of times, and then just thought, I suppose it really was more down to me not feeling comfortable, or ready to sort of talk about it.”* (Employee 5)

*“And I was dubious as to what her response would be, which is one of the reasons why I was holding back until it reached a point where I had to say something because I just couldn't carry on.”* (Employee 7)

*“I probably have a bit of a thing where I'm like, I don't want to talk to you about the personal stuff that's going on in my life because I think you're going to perceive me differently.”* (Employee 6)

These findings suggest that disclosure within workplace settings is not a neutral act. Participants described carefully considering who they could trust, how information might be interpreted, and whether disclosure could influence future workplace relationships. The willingness to seek support therefore appeared to depend not only on the availability of MHFA but also on the perceived psychological safety of the workplace environment. In this sense, engagement with MHFA was closely tied to broader relational and cultural dynamics within organisations.

The experience of not feeling ready to cope with anticipated reactions to disclosure might be based on the internalised stigma relating to stereotypes about mental ill-health in society. It may, of course, be based on previous experiences and encounters with colleagues. Previous studies have reported a mixed view, with diverse reactions to mental health disclosures being reported; this includes being treated more positively, reports of discrimination post disclosure and employers holding a negative attitude (Brouwers et al., 2020). Beliefs about negative stereotypes about mental illness being commonplace and inevitable strongly predict further psychological distress (Livingston & Boyd, 2010). Furthermore, perceived discrimination significantly heightens stress responses and encourages non-participation in healthy behaviours like help-seeking for mental health concerns (Pascoe & Smart Richman, 2009; Toth et al., 2021).

MHFAiders in the workplace also reported issues with the risks associated with disclosure, which contributed to reduced engagement of employees with MHFAiders. Their accounts highlighted the interpersonal dynamics surrounding disclosure, particularly the influence of workplace hierarchies and how these structures can discourage open communication about mental health difficulties. In most cases, for example, employees were very concerned that the fact of disclosure would become known to line managers and how this would shape perceptions of their performance:

*Because I think they would worry that it would get back to their manager. And depending on the relationship they already have with their manager, that could present more of a problem for that individual, then be, then it'd be beneficial.* (First aider 5)

*I think there's a stigma. And that I think that centres around my mental health will affect my performance. And I don't want people to know that my performance isn't that good because of my mental health. And I think that's in a corporation; I think that's probably a big barrier.* (First aider 8)

Participants’ relationships with managers appear to be key to an understanding of the reasons for disclosure or non-disclosure. Not surprisingly, a good relationship with the manager has been identified as a primary intrinsic motivating factor for disclosing mental health issues (Dewa et al., 2021). Furthermore, employees who feel valued and supported by their managers are more likely to experience job satisfaction and lower levels of anxiety and depression Gilbreath *& Benson, 2004. As pointed out by an employee (Employee 4), the culture of support available appears quite unpredictable and heavily depends upon the interpersonal relationships and dynamics between employees and line managers:

*if you've got a really good manager, then you've got a lot more support than somebody who sits right next to you in the office who has got a different manager who isn't supportive and understanding.*

The above extracts point to significant barriers relating to disclosure decision processes: Who do employees feel comfortable talking to, and what influences the disclosure process? Interpersonal relationships within the workplace appear pivotal to the disclosure process and yet they are not adequately addressed anywhere in mental health interventions. Participants' accounts indicate that disclosure within workplace settings was often perceived as a calculated risk rather than a straightforward act of help-seeking. Decisions to disclose were influenced by concerns regarding confidentiality, workplace relationships, professional reputation, and potential future consequences. These findings suggest that perceptions of psychological safety may play a critical role in determining whether employees engage with MHFA support.

Furthermore, a lack of attention to workplace cultures highlights how systemic problems, such as a lack of trust between employees and managers, can serve as obstacles to the successful implementation of mental health interventions, and thus help-seeking. It appears that for MHFA to thrive in a workplace, good interpersonal relationships with colleagues, based on trust and the assurance of anonymity, need to be mandatory for any chance of success. This is consistent with a study on the disclosure dilemma undertaken by Toth and colleagues (2021), who found that a positive relationship with the supervisor was important to the disclosure process.

Therefore, if MHFA is still implemented in the workplace, cultural issues that encourage employees to doubt the trustworthiness of potential people to disclose to must first be addressed before there is any hope that any such interventions might succeed.

## Discussion

The current study explored employees' experiences of how MHFA is implemented and functions in the workplace. The analytical themes identified from the data were a) Ambiguity surrounding the purpose of MHFA, b) Uncertainty and scepticism about the role and effectiveness of MHFA support. (subthemes: Anticipated Stigma, MHFA not addressing core concerns affecting staff well-being) and c) Risks to personal relationships following disclosure. The qualitative findings should also be interpreted in the context of the wider EMPOWER trial programme. Although the qualitative data provide important insight into participants' experiences of MHFA, the quantitative outcomes of the trial remain under review at the time of writing. Future interpretation of these findings should therefore consider the qualitative and quantitative evidence together once the trial findings are publicly available.

Overall, participants expressed their doubts about the purpose of MHFA in supporting the mental health concerns of their own or their colleagues. Such persistent doubts appear to have impacted MHFA engagement, including concerns around anticipated stigma. In addition, some participants referred to hierarchical influences and the palpable risk related to a mental health disclosure, especially on career and other job opportunities. Previous studies exploring barriers to implementing mental health and well-being initiatives in the workplace have reported similar findings (Joanna et al., 2022; Mellor & Webster, 2013; Quirk et al., 2018).

Communication of the organisations’ intentions to support and engage employees in mental health and well-being interventions is critical to the success of any mental health initiatives (Mattke et al., 2020). There is a recognised need to employ strategic communication to promote effective mental health initiatives, including the need to address common perceptions regarding organisations’ lack of genuine engagement with mental health issues (Kent et al., 2016). HR practices, for example, have been shown to favour the organisation to the detriment of employee well-being (Ogbonnaya et al., 2017). Although participants recognised the value of MHFA, they consistently evaluated it within the context of broader organisational commitment to employee wellbeing. Positive perceptions of MHFA were strongest where participants believed it formed part of a genuine organisational strategy rather than a standalone wellbeing initiative. This interpretation aligns with previous research demonstrating that visible organisational commitment supports employee wellbeing and engagement (Harvey et al., 2014; Robertson-Hart, 2020).

The study highlights continuing ambiguity regarding the purpose and boundaries of MHFA, uncertainty about the support employees can expect, and concerns about disclosure within organisational hierarchies. Together, these findings suggest that organisations should communicate more clearly how MHFA fits within wider mental health support pathways, including the limits of the role, confidentiality arrangements, and available organisational support. On the other hand, some participants viewed MHFA as a meaningful addition to existing workplace wellbeing provision, particularly compared with previous initiatives perceived as less visible or accessible. Participants frequently valued the availability of trained colleagues, opportunities for non-judgemental conversations, and increased awareness of mental health within the workplace. For some, MHFA represented a tangible demonstration of organisational commitment to employee wellbeing and contributed to greater confidence in discussing mental health concerns. Although they also identified important limitations relating to role ambiguity, disclosure concerns, and organisational stressors, the findings also indicate that MHFA can contribute positively to workplace mental health cultures when implemented within supportive organisational environments.

To effectively communicate the role of MHFA within an organisation, it is essential to clarify its capabilities and limitations. MHFA is designed to provide immediate support to individuals experiencing mental health difficulties and to guide them toward appropriate professional services; it is not intended to replace formal mental health care or therapeutic intervention (Kitchener & Jorm, 2002). Its role should therefore be considered within the wider organisational context shaping employee wellbeing. Herzberg's Two-Factor Theory provides a useful post-hoc interpretive lens for these findings. Participants consistently distinguished between support aimed at helping employees manage distress and organisational action to address the conditions contributing to that distress. While MHFA was valued as an important source of support, participants questioned its capacity to compensate for organisational factors such as workload, psychological safety and management practices. These findings suggest that MHFA is likely to be most effective when implemented alongside broader organisational strategies that address structural determinants of employee wellbeing.

Furthermore, linking MHFA initiatives directly to organisational management practices is crucial for fostering a supportive workplace culture. Organisations should integrate MHFA into their overall health and safety strategies, demonstrating a commitment to managing mental health risks alongside physical health. Identifying workplace factors that may contribute to mental health issues, such as workload, job demands, and interpersonal relationships, should be part of this overall strategy. By establishing clear connections between MHFA and broader workplace policies, organisations can promote a systemic approach to mental well-being, ensuring that employees see MHFA as part of their organisation's efforts to cultivate a mentally healthy work environment.

Investing in better training for managers engaging with MHFA is vital for maximising its effectiveness. Training programmes should focus on developing skills that allow managers to support their teams proactively, including active listening, empathy, and signposting to appropriate resources. Additionally, these training sessions should align with the Health and Safety Executive (HSE) Management Standards (Health & Safety Executive (HSE), 2023), which outline factors that can influence mental well-being in the workplace. By equipping managers with the knowledge and tools to engage with MHFA effectively, organisations can enhance their capacity to address mental health issues, foster open communication, and ultimately create a more resilient workforce. These findings also highlight the importance of equipping managers to support MHFA implementation and foster psychologically safe workplaces.

The findings suggest that MHFA should not be understood solely through the lens of barriers or effectiveness. Participants simultaneously described meaningful benefits associated with increased awareness, support, and workplace dialogue, while also highlighting limitations relating to organisational context and structural determinants of wellbeing. This tension underscores the importance of understanding MHFA as one component of a broader organisational approach to workplace mental health.

### Strengths and limitations

Several limitations should be considered when interpreting these findings. First, the number of Mental Health First Aiders who actively used their training to support colleagues was relatively small, reflecting the early stage of MHFA implementation within participating organisations. Similarly, only a limited number of employees reported receiving support from a Mental Health First Aider during the study period, which constrained the pool of potential interviewees. As a result, stakeholder groups were unevenly represented, with more MHFAiders than recipients participating. The recipient sample also added complexity: four of the five recipients had completed MHFA training, so their accounts were shaped by both recipient and MHFAider perspectives. While focusing on their role as recipients, we acknowledge that their experiences of both receiving and providing MHFA support may have influenced their accounts.

Additionally, only one senior manager participated, limiting insight into managerial perspectives. Although organisational culture emerged across participant groups, future studies should recruit a broader range of organisational leaders to examine managerial experiences of MHFA implementation.

All interviews were remote, conducted via Microsoft Teams. This method facilitated participation across geographically dispersed organisations and reflected workplace realities during and after COVID-19. However, it may have limited observations of non-verbal cues and been affected by technical issues or distractions, potentially impacting the depth of disclosures. Another limitation involves voluntary participation; Voluntary participation may have resulted in overrepresentation of participants with particularly positive or negative experiences of MHFA. Although the dataset included both supportive and critical accounts, employees with more neutral or limited experiences may be underrepresented.

Despite these potential limitations, studies have shown that conducting interviews via Teams can facilitate effective and efficient data collection (Sah et al., 2020). Researchers addressed these limitations by implementing several mitigation strategies. Specifically, the study included pilot interviews and clear guidance for participants on minimising environmental distractions and keeping their cameras on to facilitate the observation of non-verbal cues. The study explored the effect of MHFA within a workplace context, and the qualitative interviews assisted with an in-depth examination of individuals’ lived experience of MHFA; to better understand factors contributing to its uptake, or otherwise. It was evident from the study that organisations need to make clear how their existing policies, including legal requirements around discrimination, will help support the implementation of MHFA.

## Conclusions

This study has examined the experiences of MHFA in the workplace, highlighting its potential benefits such as improved mental health literacy, enhanced confidence in supporting colleagues, and more meaningful conversations about mental health while also identifying significant challenges to its effective implementation. Key barriers include ambiguity regarding MHFA's purpose, reluctance to engage stemming from mixed feelings about its support, and concerns about personal and professional relationships following disclosure. These challenges are closely linked to workplace culture, trust, and the overall organisational environment, which can either encourage or hinder MHFA initiatives. To tackle these issues, future research should aim for larger samples of MHFA recipients to gain a better understanding of its impact over time and its interaction with organisational hierarchies. Organisations need to strategically implement MHFA by clearly communicating its purpose and limitations, fostering a stigma-free environment, and aligning it with broader health strategies, including workload management and access to professional services. Furthermore, training for managers is crucial for effectively supporting employees, as it can help clarify the purpose and boundaries of MHFA, reduce ambiguity around available support, and foster a psychologically safe environment that mitigates fears of stigma and negative consequences following disclosure. MHFA England should consider revising its training curriculum to focus on everyday mental health challenges while ensuring clear communication about the role of MHFAiders. Additionally, developing resources to promote a supportive workplace culture and advocating for stronger legal protections for employees under the Equality Act (2010) can enhance the effectiveness of MHFA. By addressing these recommendations, stakeholders can collaborate to create a healthier and more supportive work environment for all employees.

## Supplementary Material

Supplementary material V1.docx Supplementary material V1.docx

Recipients Interview Questions.pdf Recipients Interview Questions.pdf

Employee Interview Questions.pdf Employee Interview Questions.pdf

Senior Manager Interview questions.pdf

## Data Availability

The data supporting this study's findings are available from the corresponding author [OA], upon reasonable request. For the purpose of open access, the author has applied a Creative Commons Attribution (CC BY) licence to any Author Accepted Manuscript version arising.
