# Supplementary material for: Mental health first aid in the workplace: a reflexive thematic analysis of UK workers’ experiences
Source: Int J Qual Stud Health Well-being. 2026 Jul 21;21(1):2706909. doi: 10.1080/17482631.2026.2706909 (PMC13393055; doi:10.1080/17482631.2026.2706909)

**Supplementary material showing the analytical journey from code clustering to theme development.**


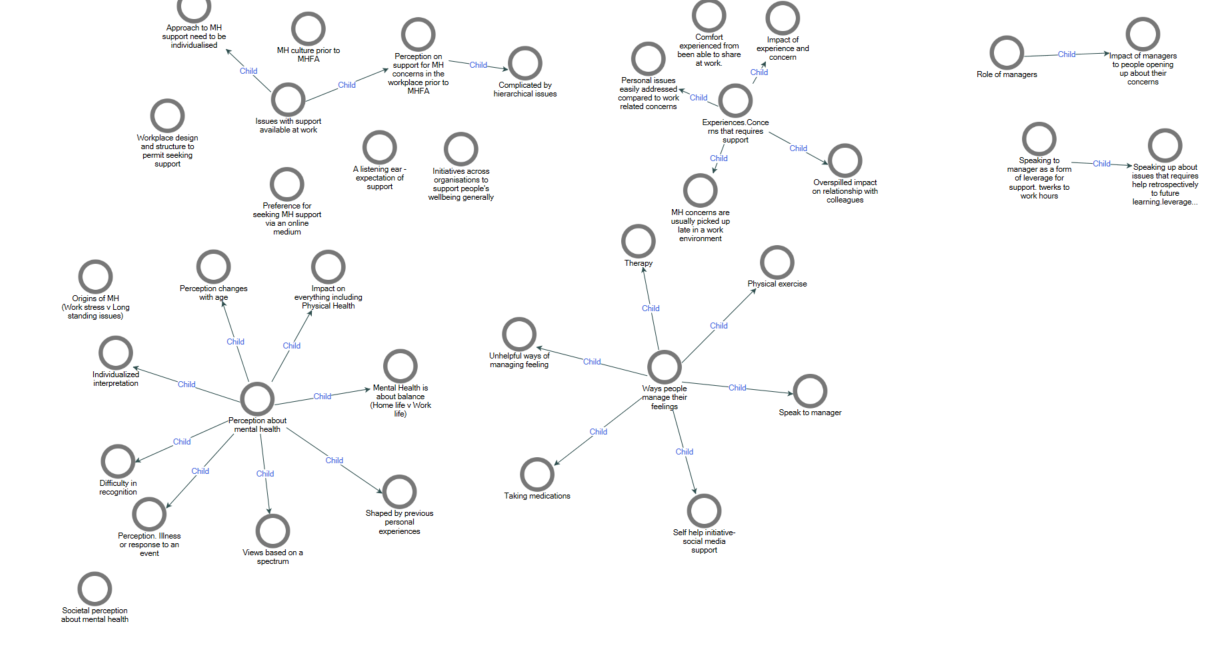


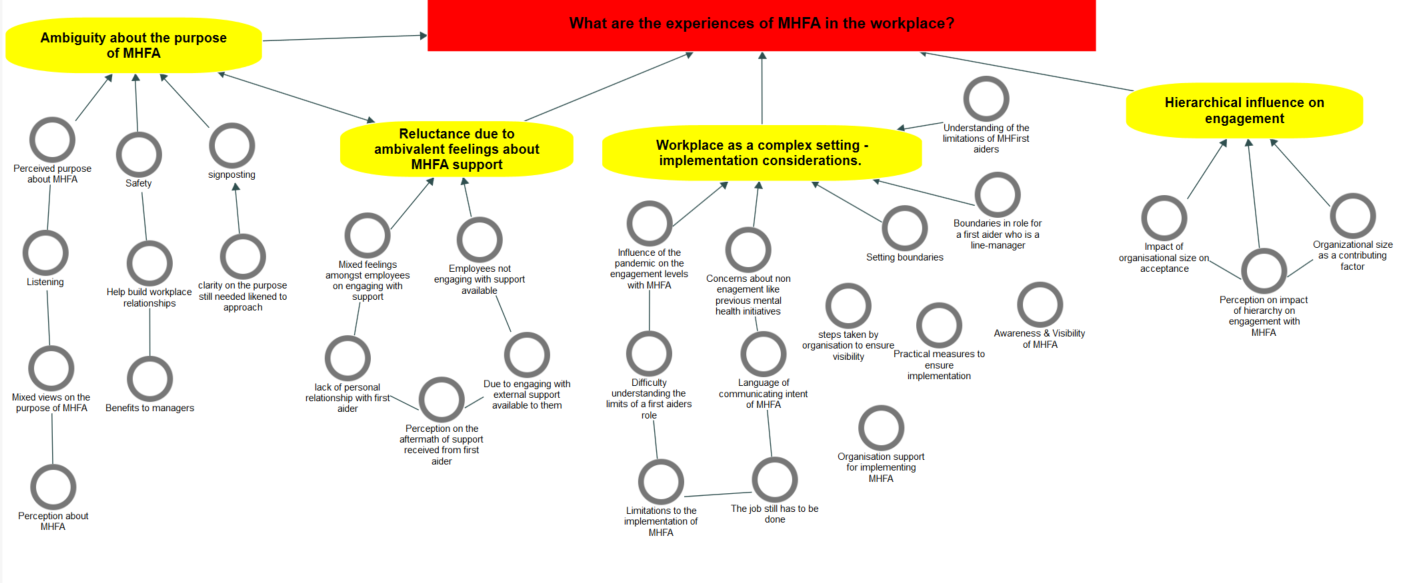


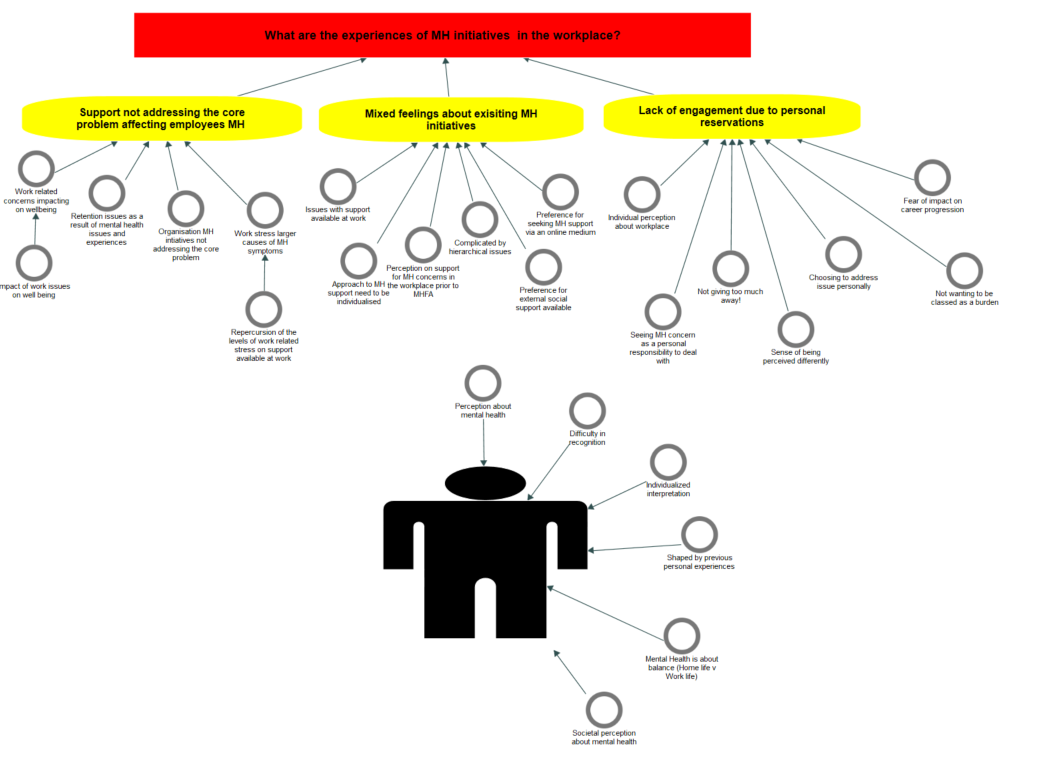

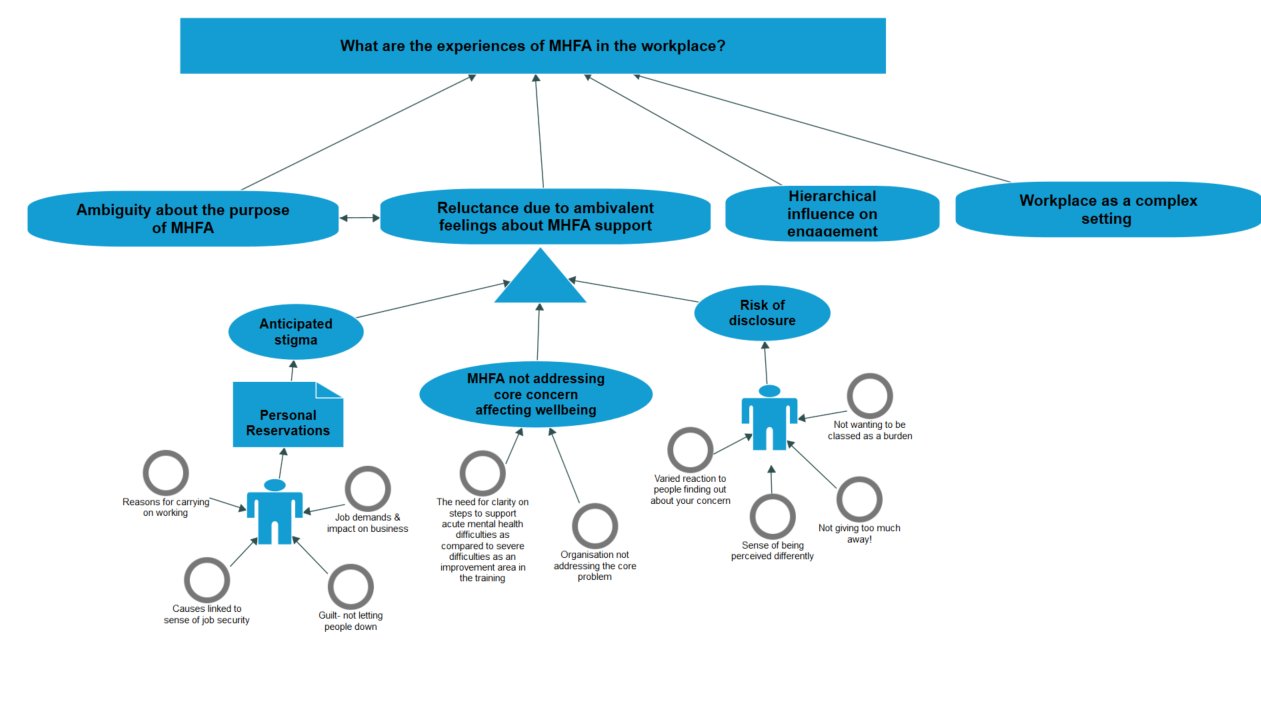

Supplement: Supplementary material V1.docx [file ZQHW_A_2706909_SM9686.docx]
