# Supplementary material for: Mental health first aid in the workplace: a reflexive thematic analysis of UK workers’ experiences
Source: Int J Qual Stud Health Well-being. 2026 Jul 21;21(1):2706909. doi: 10.1080/17482631.2026.2706909 (PMC13393055; doi:10.1080/17482631.2026.2706909)
Supplement: Recipients Interview Questions.pdf [file ZQHW_A_2706909_SM9685.pdf]

Recipients Interview Questions

- **Thank you for agreeing to speak to me.**
- **Introduce yourself.**
- **Briefly talk about the Project and what we hope to get out of the interviews today.**
- **Share the Participants information sheets and consent form with the participants for signatures.**
- **Icebreaker (Get the participants to introduce themselves and ask them how they would like to be addressed)**
- **Reassure participants about confidentiality, encouraging them to speak about their experiences freely in their own words. (Mention that any discussions that might signify any threat of danger to the participants or others would be shared with a designated member of the research as a risk management measure)**

S/N	Questions	Prompts	Objectives Covered
1.	**Can you tell me a little about the background to the issues that you shared with the mental health first aider?**	• When did they first arise?<br>• How did they develop over time?<br>• What led to their decision to share at work?<br>• What led to the decision to the approach MHFA more specifically?	• Any perceived changes in relationships at work because of their mental health challenge, as well as the support they have received from the MHFA trained staff. This would include relations across the spectrum, such as relations with line managers as well as relations with equal level colleagues
2.	**After you made the decision to approach the**	• Explore what sort of experiences they have shared with someone at work.	• Exploration of recipients' perceptions of the social impact

	**MHFA, what was your experience of sharing your concerns at work?**	• Ask them who they shared their experiences with? i.e. did they share with a colleague and then an MHFA or did they go straight to the MHFA?<br>• Explore how they felt about these experiences and how they managed their feelings.<br>• Explore the barriers to sharing their concerns/experiences at work.	through the lens of their social well-being: social integration, social acceptability, social contribution, social actualization, and social coherence.
3.	**Following the concerns discussed earlier, do you consider these issues to be linked to your mental health? If so, what is your understanding of how they are linked?**	• Explore their understanding of mental health.<br>• Explore whether they think the mental health issues are due to long standing issues and/or life-events or are more specifically related to work stresses.<br>• Do they think their 'well-being' is compromised by work issues?<br>• How do they think about mental health issues? As an illness or a response to events that have occurred…? Or both…?<br>• Were there any barriers in being able to talk about any of these concerns?	• Exploration of recipients' perceptions of the social impact through the lens of their social well-being: social integration, social acceptability, social contribution, social actualization, and social coherence.<br>• The perceived quality of support for mental health challenges from the MHFA trained staff – here, we would explore with the participant in-depth the nature and quality of the help received, as well as more generic aspects

			of their experience, such as warmth, empathy etc.
4.	**After you had spoken to the MHFA, how would you describe the kind of support you received and the ways in which it affected your feelings and your mental health?**<br>**(Note to researcher: With regards to daily work? Community? Family?)**<br>**(Encourage them to give examples)**	• Explore the support available both at work & outside work.<br>• Ask them to describe what specific qualities the MHFA was able to deliver – this should be quite detailed, so ask for examples and particular incidences of positive and/or negative.<br>• Ask them about any other forms of support they received (in addition to the MHFA). (At work or Outside work) (Formal or Informal)<br>• Explore the impact of the pandemic on the sort of support that was available. (At work or outside work)<br>• Find out how helpful the support they explored. (Encourage them to give examples).<br>• Explore the impact of the pandemic on how they manage their feelings.	•The perceived quality of support for mental health challenges from the MHFA trained staff – here, we would explore with the participant in-depth the nature and quality of the help received, as well as more generic aspects of their experience, such as warmth, empathy etc.
5.	**Let us talk about your encounter with your MHF-Aider, what are your**	• Explore how helpful MHFA was.<br>• Explore how they felt about their encounter with the MHF-Aider. (Safety, warmth, confidentiality)	•The perceived quality of support for mental health challenges from the MHFA trained staff – here, we would explore with the

	**thoughts on the help you received?**<br><br>**How did the opportunity come about?**<br>**What do you think is the purpose of MHFA?**	• Explore the impact of the help received from the MHF-Aider on their concerns raised earlier.<br>• Explore further the impact on relationships at work?	participant in-depth the nature and quality of the help received, as well as more generic aspects of their experience, such as warmth, empathy etc.
6.	**Before we roundup, let's talk about *presenteeism*. What is your understanding of presenteeism? That is people being at work despite not feeling their best or not feeling able to work.**<br><br>**(Encourage them to give examples of instances where they have attended work whilst feeling unwell)**	• Explore if their state of being unwell was mental health or physical health related.<br>• Explore the reasons for carrying on with work.<br>• Explore how they feel about carrying on with work despite being unwell.<br>• Explore the potential contributory factors to carrying on with work.<br>• Explore how the difficulty experienced when trying to be open impact on carrying on with work.<br>• In your view, is there any benefit from presenteeism (to the individual to the organisation)?	• Any significant changes to workplace relations and organizational behavior because of seeking help for mental health challenges.<br>• Their perceptions of relations outside of work, including an improvement in being able to discuss mental health with others.<br>• Exploration of recipients' perceptions of the social impact through the lens of their social well-being: social integration, social acceptability, social

			contribution, social actualization, and social coherence.
7.	**Is there anything you were expecting to discuss that we didn't talk about? (Use this opportunity to touch on the questions that were not properly explored)**		

- **Conclude the interview by explaining to the participant that quotes from the interviews could appear in publications but would not be identifiable.**
- **Thank you for your time**.
