# Supplementary material for: Mental health first aid in the workplace: a reflexive thematic analysis of UK workers’ experiences
Source: Int J Qual Stud Health Well-being. 2026 Jul 21;21(1):2706909. doi: 10.1080/17482631.2026.2706909 (PMC13393055; doi:10.1080/17482631.2026.2706909)
Supplement: Employee Interview Questions.pdf [file ZQHW_A_2706909_SM9687.pdf]

Employee Interview Questions

- **Thank you for agreeing to speak to me.**
- **Introduce yourself.**
- **Briefly talk about the Project and what we hope to get out of the interviews today. Share the Participants information sheets and consent form with the participants for signatures.**
- **Get the participants to introduce themselves and ask them how they would like to be addressed**
- **Reassure participants about confidentiality, encouraging them to speak about their experiences freely in their own words. (Mention that any discussions that might signify any threat of danger to the participants or others would be shared with a designated member of the research as a risk management measure)**
- **Start with the questions**

S/N	Questions	Prompts	Objectives covered
1	**Let us start off with what it is like sharing your concerns/experiences at work?**	• Explore the sort of difficult experiences that they would have loved to share with someone at work.<br>• Explore how they felt about these experiences and how they managed their feelings.<br>• Explore the barriers to sharing their concerns/experiences at work.	• Exploration of recipients' perceptions of the social impact through the lens of their social well-being: social integration, social acceptability, social contribution, social actualization, and social coherence.<br><br>• Any perceived changes in relationships at work because of their mental health challenge, as well as the support they have received from the MHFA trained staff. This would include relations across the spectrum, such as relations with line managers as well

			as relations with equal level colleagues
2	**Following the concerns discussed earlier, do you consider these concerns linked to your mental health? If so, what is your understanding of how they are linked?**	• Explore their understanding of mental health.<br>• Explore whether they think the mental health issues are due to long standing issues and/or life-events or are more specifically related to work stresses.<br>• Do they think their 'well-being' is compromised by work issues?<br>• How do they think about mental health issues? As an illness or a response to events that have occurred…? Or both…?<br>• Were there any barriers in being able to talk about any of these concerns?	Exploration of recipients' perceptions of the social impact through the lens of their social well-being: social integration, social acceptability, social contribution, social actualization, and social coherence.
3	**You talked about the way you felt about this concerns earlier, how did you manage those feelings and the other support you explored?**<br>**(Note to researcher: With regards to daily work? Community? Family?) (Encourage them to give examples)**	• Explore the support available both at work & outside work.<br>• Ask them about any other forms of support they received (At work or Outside work) (Formal or Informal)<br>• Explore the impact of the pandemic on the sort of support that was available. (At work or outside work)<br>• Find out how helpful the support they explored. (Encourage them to give examples).<br>• Explore the impact of the pandemic on how they manage their feelings.	The perceived quality of support for mental health challenges from the MHFA trained staff – here, we would explore with the participant in-depth the nature and quality of the help received, as well as more generic aspects of their experience, such as warmth, empathy etc.

**4**	**Reflecting on the introduction of MHFA in your organization, what is the purpose of MHFA?**	• Explore what has changed since the introduction.<br>• Explore how visible are the opportunities to get help from a trained MHF-Aiders.<br>• Explore any impact on current work climate.<br>• Explore room for improvements in the implementation of this new initiative.	The perceived quality of support for mental health challenges from the MHFA trained staff – here, we would explore with the participant in-depth the nature and quality of the help received, as well as more generic aspects of their experience, such as warmth, empathy etc.
**5**	**Before we roundup, lets talk about presenteeism. What is your understanding of presenteeism?**<br><br>**(Encourage them to give examples of instances where they have attended work whilst feeling unwell)**	• Explore if their state of being unwell was mental health or physical health related.<br>• Explore the reasons for carrying on with work.<br>• Explore how they feel about carrying on with work despite being unwell.<br>• Explore the potential contributory factors to carrying on with work.<br>• Explore how the difficulty experienced when trying to be open impact on carrying on with work.<br>• In your view, is there any benefit from presenteeism (to the individual to the organisation)?	• Any significant changes to workplace relations and organizational behavior because of seeking help for mental health challenges.<br>• Their perceptions of relations outside of work, including an improvement in being able to discuss mental health with others.<br>• Exploration of recipients' perceptions of the social impact through the lens of their social well-being: social integration, social acceptability, social contribution, social actualization, and social coherence.
**6**	**Is there anything you were expecting to be discussed that was not discussed during the course of the interview? (Use this opportunity to touch on the**		

	**questions that were not properly explored)**		

- **Conclude the interview by explaining to the participant that quotes from the interviews could appear in publications but would not be identifiable.**
- **Thank you for your time**.
