# Supplementary material for: Mental health first aid in the workplace: a reflexive thematic analysis of UK workers’ experiences
Source: Int J Qual Stud Health Well-being. 2026 Jul 21;21(1):2706909. doi: 10.1080/17482631.2026.2706909 (PMC13393055; doi:10.1080/17482631.2026.2706909)
Supplement: Senior Manager Interview questions.pdf [file ZQHW_A_2706909_SM9688.pdf]

Senior Manager’s Interview Questions

- **Thank you for agreeing to speak to me.**
- **Introduce yourself.**
- **Briefly talk about the Project and what we hope to get out of the interviews today. Share the Participants information sheets and consent form with the participants for signatures.**
- **Icebreaker (Get the participants to introduce themselves and ask them how they would like to be addressed)**
- **Reassure participants about confidentiality, encouraging them to speak about their experiences freely in their own words. (Mention that any discussions that might signify any threat of danger to the participants or others would be shared with a designated member of the research as a risk management measure)**
- **Start with the questions.**

S/N	Questions	Prompts	Objectives covered
**1.**	**Can you tell me a bit about how MHFA was introduced in your organisation?**	• **How did you hear about it?**<br>• **Did you understand who it was for? And what it was for?**<br>• Explore what has changed since the introduction.<br>• Explore how visible are the opportunities to get help from a trained MHF-Aiders.<br>• Explore any impact on current work climate.<br>• Explore room for improvements in the implementation of this new initiative.	The perceived quality of support for mental health challenges from the MHFA trained staff – here, we would explore with the participant in-depth the nature and quality of the help received, as well as more generic aspects of their experience, such as warmth, empathy etc.

**2.**	**Considering your role as a senior manager, can you share some thoughts on the implementation of MHFA in your workplace?**	• Explore the challenges faced whilst trying to implement MHFA.<br>• Explore the recruited and selection strategy adopted in getting employees trained.<br>• Explore the support given to the first aiders following the training.<br>• Explore the impact of the intervention on the culture within the workplace.<br>• Explore any challenges currently been faced in the implementation of MHFA.<br>• Explore other areas they would like to see changes to augment the current effort in implementing MHFA	Any significant changes to workplace relations and organizational behaviour because of seeking help for mental health challenges.
**3.**	**Before we roundup, let's talk about *presenteeism*. What is your understanding of presenteeism?**<br><br>**(Encourage them to give examples of instances where they have attended work whilst feeling unwell)**	• Explore if their state of being unwell was mental health or physical health related.<br>• Explore the reasons for carrying on with work.<br>• Explore how they feel about carrying on with work despite being unwell.<br>• Explore the potential contributory factors to carrying on with work.<br>• Explore how the difficulty experienced when trying to be open impact on carrying on with work.	• Any significant changes to workplace relations and organizational behavior because of seeking help for mental health challenges.<br>• Their perceptions of relations outside of work, including an improvement in being able to discuss mental health with others.

		• In your view, is there any benefit from presenteeism (to the individual to the organisation)?	• Exploration of recipients' perceptions of the social impact through the lens of their social well-being: social integration, social acceptability, social contribution, social actualization, and social coherence.
**4.**	**Is there anything you were expecting to be discussed that we did not talk about? (Use this opportunity to touch on the questions that were not properly explored)**		

• Conclude the interview by explaining to the participant that quotes from the interviews could appear in publications but would not be identifiable.

• Thank you for your time.
